# Inflammation and daily memory lapses among older adults in the Einstein Aging Study: Associations by mild cognitive impairment status and gender

**DOI:** 10.1002/alz.089565

**Published:** 2025-01-09

**Authors:** Erin E. Harrington, Karina Van Bogart, Jennifer E. Graham‐Engeland, Jacqueline A. Mogle, Mindy J. Katz, Richard B. Lipton, Martin J. Sliwinski, Christopher G. Engeland

**Affiliations:** ^1^ University of Wyoming, Laramie, WY USA; ^2^ The Pennsylvania State University, University Park, PA USA; ^3^ Clemson University, Clemson, SC USA; ^4^ Albert Einstein College of Medicine, Bronx, NY USA; ^5^ Pennsylvania State University, State College, PA USA

## Abstract

**Background:**

Inflammation is a risk factor for cognitive decline, mild cognitive impairment (MCI), and Alzheimer’s disease (AD). While past research in laboratory settings suggests that inflammation relates to cognitive decline and MCI status, more research is needed to examine such associations in everyday life. The present work addressed this gap by examining MCI and gender stratified links between circulating inflammatory biomarkers and self‐reported prospective memory (PM; i.e., memory for future events) and retrospective memory (RM; i.e., memory for past events/information) lapses measured at the end of each day via daily dairies.

**Method:**

Older adults (n=270, Mage=76.97, 68% female) enrolled in the Einstein Aging Study were classified with or without MCI using Jak/Bondi criteria. Participants completed a two‐week protocol, including two blood draws (at the start and end of the protocol) from which circulating (basal) cytokines were quantified (interleukin [IL]‐1b, IL‐4, IL‐6, IL‐8, IL‐10, tumor necrosis factor‐alpha [TNF‐α]). Each night, participants completed reports of PM and RM lapses that had occurred that day via smartphones. Correlations between inflammatory markers (averaged across the two draws) and memory lapses stratified by MCI status and gender were estimated.

**Results:**

Among those with MCI, men (n=23) exhibited higher levels of circulating IL‐10 in association with more frequent PM lapses (r=.47, p=.025) and women (n=55), exhibited higher circulating IL‐6 in association with more frequent RM lapses (r=.28, p=.035). Among those without MCI higher levels of IL‐8 correlated with more frequent PM lapses only in men (n=64; r=.39, p=.002). Findings held when controlling for BMI, age, and education.

**Conclusion:**

The present research suggests that MCI‐ and gender‐dependent links between inflammation and everyday memory lapses may be restricted to certain inflammatory cytokines. IL‐10 and IL‐6 may play a role in MCI‐related daily forgetting among men and women, respectively. However, these findings should be replicated within a larger sample of individuals experiencing MCI. Additionally, longitudinal research is needed to determine whether IL‐8 is indicative of early cognitive decline prior to MCI in men. Longitudinal examinations will better elucidate the links between inflammation, memory lapses, and MCI, and might ultimately inform early risk detection for MCI and AD.

